# Dietary Branched-Chain Amino Acid Intake Is Associated with Muscle Mass and Handgrip Strength: Evidence from China—Health and Nutrition Survey 2015–2024

**DOI:** 10.3390/nu18101546

**Published:** 2026-05-13

**Authors:** Zhihan Xu, Yifei Ouyang, Chang Su, Jiguo Zhang, Wenwen Du, Xiaofang Jia, Yuehui Fang, Yiyao Lian, Feifei Huang, Li Li, Jing Bai, Yanli Wei, Xiaofan Zhang, Fangxu Guan, Huijun Wang, Yuna He

**Affiliations:** 1 National Institute for Nutrition and Health, Chinese Center for Disease Control and Prevention, Beijing 100050, China; xzh834126998@163.com (Z.X.); ouyyf@ninh.chinacdc.cn (Y.O.); suchang@ninh.chinacdc.cn (C.S.); zhangjg@ninh.chinacdc.cn (J.Z.); duww@ninh.chinacdc.cn (W.D.); jiaxf@ninh.chinacdc.cn (X.J.); fangyh@ninh.chinacdc.cn (Y.F.); lianyy@ninh.chinacdc.cn (Y.L.); huangff@ninh.chinacdc.cn (F.H.); lili@ninh.chinacdc.cn (L.L.); baijing@ninh.chinacdc.cn (J.B.); weiyl@ninh.chinacdc.cn (Y.W.); zhangxf@ninh.chinacdc.cn (X.Z.); guanfx@ninh.chinacdc.cn (F.G.); wanghj@ninh.chinacdc.cn (H.W.); 2 Key Laboratory of Public Nutrition and Health, National Health Commission of the People’s Republic of China, Beijing 100050, China

**Keywords:** branched-chain amino acids, appendicular skeletal muscle mass, handgrip strength, mixed-effect model

## Abstract

Objectives: This study aims to investigate the associations between dietary branched-chain amino acids (BCAAs) intake and appendicular skeletal muscle mass (ASM) as well as handgrip strength in Chinese adults. Methods: A total of 36,086 observations (54.32 ± 14.63 y) were included from the China Health and Nutrition Survey (CHNS) across three waves (2015, 2018, and 2022–2024). ASM was measured by bioelectrical impedance analysis, and handgrip strength was measured using a digital dynamometer. Dietary BCAA intake was assessed using three consecutive 24 h dietary recalls and adjusted for energy intake. Multilinear mixed-effect models were employed to examine the longitudinal association between BCAA intake and ASM. Multivariable regression was used to assess the cross-sectional association between BCAA intake and handgrip strength. Results: Dietary BCAA intake was significantly associated with ASM (β = 0.074, *p* < 0.05) with adjustment for potential confounding factors. This estimated positive effect increased with age in both males and females, and was consistently stronger in males. Compared with the lowest quintile (Q1), Q4 of dietary BCAA intake had higher handgrip strength (β = 0.721, *p* < 0.001). Stratified analyses showed that this association was more pronounced in males (Q4 vs. Q1: β = 1.016, *p* = 0.005) and in participants aged ≥65 years (Q4 vs. Q1: β = 1.024, *p* = 0.008). Conclusions: Dietary BCAA intake is recommended to maintain muscle mass and strength in Chinese adults.

## 1. Introduction

Sarcopenia is defined as an age-related decline in skeletal muscle mass, strength and function, which is strongly associated with a range of adverse health outcomes, including increased risk of falls, functional impairment, hospital admission, cardiometabolic disease, dementia and all-cause mortality [1]. Formal recognition of sarcopenia with a designated International Classification of Diseases, Tenth Revision, Clinical Modification (ICD-10-CM) code (M62.84) marked a significant milestone in establishing its clinical significance and promoting awareness within the medical community [2]. The Asian Working Group for Sarcopenia (AWGS) introduced the first diagnostic consensus in 2014 [3] and updated it in 2019 [4]. A meta-analysis of sarcopenia epidemiology in Asia, utilizing the AWGS 2019 diagnostic criteria, showed that the overall prevalence of sarcopenia among community-dwelling older adults was 16.5% (95% CI: 14.7–18.4%) [5]. In 2025, AWGS simplified the diagnostic algorithm to require only ASM and handgrip strength [6]. The dual-energy X-ray absorptiometer (DEXA) and bioelectrical impedance analysis (BIA) are most common in assessment of ASM. A recent study has also emphasized the clinical value of simple and feasible approaches for estimating ASM in older adults, using an equation that includes the body weight of the patient as the sole variable [7]. Given that China is one of the countries with the fastest-growing aging population in the world, and considering emerging evidence indicating that sarcopenia is not solely confined to older adults (muscle mass potentially begins to decline as early as age 45) [8], maintaining muscle health has become a significant public health priority.

Branched-chain amino acids (BCAAs; valine, leucine, and isoleucine) are primarily metabolized and utilized as energy sources within skeletal muscle tissue [9]. In addition, BCAAs, especially leucine, function as critical signaling molecules that promote muscle protein synthesis through activation of the mammalian target of the rapamycin complex 1 (mTORC1) pathway [10]. Beyond muscle mass, experimental evidence suggests that BCAAs may also contribute to muscle function by supporting skeletal muscle energy metabolism and mitochondrial biogenesis, which are essential for muscle contraction and physical performance [11]. A meta-analysis of 35 randomized controlled trials (RCTs) revealed that BCAAs exert beneficial effects on muscle mass, strength and physical performance in older adults [12]. A cross-sectional study involving over 100,000 participants observed positive correlations between circulating BCAA levels and both muscle mass and muscle strength [13]. Furthermore, research conducted on community-dwelling older adults in China indicates that a higher dietary BCAA intake was significantly associated with lower prevalence of sarcopenia [14].

Although studies have examined the associations of BCAAs with muscle-related outcomes, most previous evidence has focused on circulating BCAA levels, BCAA supplementation, or relatively small study populations. Evidence based on dietary BCAA intake from large-scale Chinese populations remains limited. In addition, few studies have simultaneously evaluated muscle mass and handgrip strength, which represent different aspects of sarcopenia-related muscle health. Therefore, utilizing extensive longitudinal data from CHNS 2015–2024, this study aims to investigate the associations between dietary BCAA intake and ASM as well as handgrip strength in Chinese adults.

## 2. Materials and Methods

The CHNS is a longitudinal household-based survey with a multistage, stratified sampling design to examine the socio-demographic characteristics, diet, health status, physical activity, and behaviors of the Chinese population. Comprehensive details about the survey methodology are available in the cohort file [15]. This study utilized the data collected during the periods of 2015, 2018 and 2022–2024, which is an open cohort with repeated measures. This study included 36,086 observations involving 21,228 participants aged 18 years and older. The exclusion criteria comprised: (1) missing dietary data; (2) missing data on physical measurements.

Detailed dietary intake information was collected through three consecutive 24 h dietary recalls, covering all meals and snacks for each participant. This data was obtained via face-to-face interviews conducted over two weekdays and one weekend day [16]. The BCAA content of foods was determined using the China Food Composition Table [17]. Missing items in the food composition database were imputed using values from comparable foods. The total intake of BCAA for each participant was calculated by summarizing the BCAA content multiplied by the amount of each food consumed. To enable comparison among participants with varying energy intakes, the dietary BCAA intake was adjusted to reflect the intake per 1000 kcal, with the unit being grams per day per 1000 kcal (g/d/1000 kcal). The proportion of not meeting estimated average requirements (EAR) for valine, leucine, and isoleucine were reported. The sources of BCAA were sorted into: (1) cereals and tubers; (2) meat and poultry; (3) eggs; (4) dairy products; (5) legume; (6) others.

Muscle mass was measured across all three surveys, whereas handgrip strength was evaluated exclusively in the 2022–2024 survey. The muscle mass measurement was conducted using BIA (TANIATA, BC-601, Tokyo, Japan) [18]. ASM was calculated by summing the skeletal muscle in the arms and legs. The maximum handgrip strength was measured using a digital dynamometer (Camry-EH101, Zhongshan Camry Electronic Co., Ltd., Zhongshan, China). Measurements for both the left and right hands were recorded twice, with at least a five-minute interval between each measurement. The study reported and analyzed the mean value across both hands and both measurements.

Demographic characteristics were collected through interviews conducted by a trained interviewer. The covariates assessed included age, gender (male or female), residence (urban or rural), education level (middle school or below, high school graduation and college or above), alcohol use (yes or no), smoking (never, quit or current smoker), physical activity and type 2 diabetes mellitus (T2DM). Physical activity was quantified as the average metabolic equivalents of task (MET) hours per week, encompassing four PA domains: occupational, household, travel, and leisure time activities [18]. According to diagnostic criteria recommended by the World Health Organization in 1999, T2DM was diagnosed by any one of the following criteria: (1) fasting plasma glucose (FPG) ≥ 7.0 mmol/L; (2) HbA1C ≥ 6.5; (3) self-reported history of diabetes diagnoses; (4) use of antidiabetic medication [19]. All missing covariates were coded with median values for continuous variables or mode values for categorical variables.

Continuous variables exhibiting a normal distribution were presented as means ± standard deviations (SDs). Categorical variables were presented as frequencies and percentages (*n*, %). To assess participant characteristics, analyses were conducted using the Mann–Whitney U test for continuous variables and Pearson’s Chi-square test for categorical variables. Multilinear mixed-effect models were performed to examine the association between BCAA intake and ASM. We built 3 models: model 1 included BCAA intake, age, age squared, and gender; model 2 included variables in model 1 plus BMI, educational level, residence, and interaction items (BCAA × age, BCAA × age squared, and BCAA × gender); and model 3 included variables in model 2 plus alcohol use, smoking, physical activity, and BCAA × physical activity. Multivariable regression analysis was performed to evaluate handgrip strength across BCAA intake quintiles. We built 3 covariate models as well: model 1 was unadjusted; model 2 was adjusted for age, gender, BMI, educational level, and residence; and model 3 was additionally adjusted for alcohol use, smoking, and physical activity. Further analyses were conducted by stratifying participants based on age and gender. The restricted cubic spline (RCS) was employed to examine the dose–response association between BCAA intake and handgrip strength after adjusting all covariates, with 3 knots (located at the 10th, 50th and 95th percentiles). Stratified analyses were conducted by gender and age (65 years as the demarcation age of older adults). Sensitivity analyses were conducted among the participants without diabetes. A two-sided *p*-value < 0.05 was considered statistically significant. R software (version 4.5.2) was applied to conduct the statistical analysis. Multilinear mixed-effect models were analyzed by the “lmerTest” package (version 3.2-0).

## 3. Results

### 3.1. Participants Characteristics

The characteristics of the participants in CHNS across three survey waves are presented in Table 1. Based on 36,086 observations (56.81% female; 54.32 ±14.63 years), mean ASM remained stable between 2015 and 2018 (18.58 kg) before increasing significantly to 18.87 kg in 2024 (*p* < 0.001). Mean handgrip strength in 2024 was 29.66 ± 9.89 kg. Energy intake in 2024 was significantly lower than in 2015 and 2018, driven by reductions in protein and carbohydrate consumption, alongside a marked increase in fat intake (all *p* < 0.001). Notably, although mean dietary BCAA intake increased (from 5.22 to 5.48 g/d/1000 kcal), the proportion of participants not meeting EAR for all three BCAAs rose consistently.

The proportion of dietary BCAA intake from different food sources across three waves is presented in Supplementary Materials, Figure S1. It remained stable between 2015 and 2018, followed by a marked change in 2024. There was a substantial decline in the contribution from cereals and tubers. Despite this decrease, they remained the primary source of BCAA, still accounting for over 30% of total intake. In contrast, the proportion derived from meat and poultry increased considerably, gradually narrowing the gap with cereals and tubers. Additionally, a consistent upward trend was observed in contributions from eggs and dairy products, while the proportion from legumes continued to decline.

### 3.2. Mixed-Effects Model Estimates for ASM in CHNS 2015–2024

The mixed-effects model estimates for ASM are presented in Table 2. A significant positive association was observed between dietary BCAA intake and ASM when adjusting for age and gender (β = 0.070, *p* < 0.05). In the fully adjusted model, each 1 g/d/1000 kcal increase in dietary BCAA intake remained significantly associated with an additional 0.074 kg of ASM (*p* < 0.05). Age demonstrated a non-linear relationship with ASM, characterized by a negative linear term (β = −0.011, *p* < 0.05) and a significant quadratic term (*p* < 0.05). As expected, females had lower ASM than males (β = −6.562, *p* < 0.05). Among other covariates, higher BMI, higher educational level, urban residency, and higher physical activity were positively associated with ASM (all *p* < 0.05), whereas no significant associations were observed for smoking or alcohol consumption. Sensitivity analysis showed that among individuals without diabetes, the association between dietary BCAA intake and muscle mass remained stable, with a stronger protective effect (Supplementary Materials, Table S1).

**Figure 1:**
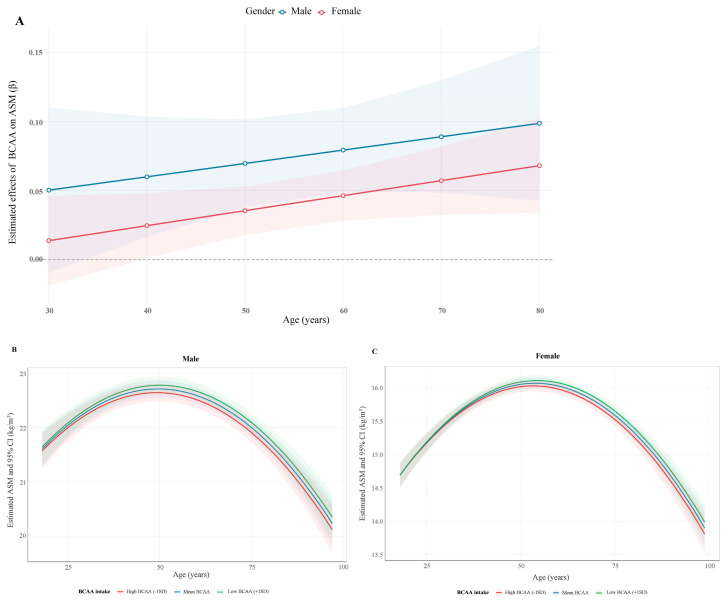
Estimated effects of BCAA intake on ASM stratified by gender and age (**A**) and estimated ASM among males (**B**) and females (**C**).

Further explored was the estimate effect of dietary BCAA intake on ASM stratified by gender and age. The protective effect of dietary BCAA intake was higher in males than in females, and increased with advancing age in both males and females (Figure 1A). Consistently, higher BCAA intake levels were associated with greater estimated ASM across all ages for both genders. The relationship between age and estimated ASM followed an inverted U-shaped pattern, with an accelerated decline beginning around age 53 (Figure 1B,C).

### 3.3. Association Between BCAA and Handgrip Strength in CHNS 2024

This multivariable analysis involved 12,060 participants from CHNS 2024. As presented in Table 3, the association between BCAA intake and handgrip strength was analyzed across different population subgroups using multivariable regression models. Among all participants, a significant positive trend was observed in Model 3. Compared to the lowest intake quintile (Q1), handgrip strength in Q4 increased by 0.721 kg (*p*< 0.001), while those in Q2 and Q3 exhibited more modest but still significant increases (0.499 kg, *p* = 0.018 and 0.439 kg, *p* = 0.039, respectively). However, no significant difference was observed in Q5 (β = 0.372, *p* = 0.09).

**Figure 2:**
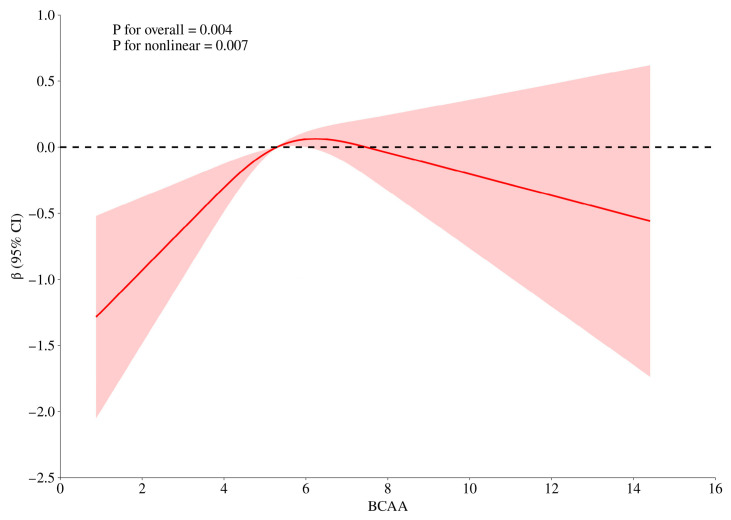
RCS analysis of association between BCAA intake and handgrip strength in CHNS 2024.

Stratified analyses revealed a significant gender-specific pattern. In males, higher BCAA intake was consistently associated with increased handgrip strength, while no significant associations were found between BCAA intake and handgrip strength across all quintiles in females. When stratified by age, significant associations were primarily evident among participants aged <65 years, with positive coefficients observed for Q2, Q3, and Q4 (all *p* < 0.05). Among participants aged ≥65 years, a significant positive association was found only for Q4 (β = 1.024, *p* = 0.008), while other quintiles showed no statistically significant relationship with handgrip strength. Restricted cubic spline (RCS) analyses revealed an inverted U-shaped nonlinear relationship between BCAA intake and handgrip strength. A daily intake of approximately 6 g/1000 kcal of BCAA was associated with the strongest positive effect on handgrip strength (Figure 2). Stratified RCS curves by gender and age also demonstrated similar patterns of association (Supplementary Materials, Figure S2). Sensitivity analysis showed that among individuals without diabetes, the inverted U-shaped association between dietary BCAA intake and handgrip strength remained significant (Supplementary Materials, Figure S3).

## 4. Discussion

Based on the data from CHNS, this study investigated the association of dietary BCAA intake with ASM and handgrip strength in Chinese adults. The findings indicate that each additional gram of BCAA intake per 1000 kcal per day was significantly associated with an increase of 0.074 kg in ASM. This beneficial effect became more pronounced with advancing age in both genders. Additionally, the association between BCAA intake and handgrip strength followed an inverse U-shaped nonlinear relationship, with an optimal intake of approximately 6 g/d/1000 kcal. This association was more consistent in males and in individuals younger than 65 years across increasing BCAA intake quintiles.

The observed associations between dietary BCAA intake and muscle mass and handgrip strength may be explained by several biological mechanisms. BCAAs, especially leucine, play an important role in skeletal muscle metabolism by activating the mTORC1 pathway and stimulating muscle protein synthesis, which may help preserve muscle mass [10]. In addition, BCAAs may serve as energy substrates in skeletal muscle and may support mitochondrial biogenesis and oxidative metabolism, which are important for muscle contraction and physical performance [11]. BCAAs were recommended by the AWGS consensus 2019 as one of nutritional supplements for hospital-based interventions for sarcopenia [4]. However, BCAA supplementation interventions were usually combined with exercise programs, which complicates the ability to isolate and determine their specific, independent protective effects on muscle health [12]. Furthermore, the protective effect of BCAA supplementation on muscles remains controversial. A meta-analysis of 35 RCTs revealed that BCAAs have beneficial effects on muscle mass, strength and physical performance in older adults [12]. The quantity of BCAA supplementation administrated among included studies was 2.5–5 g/day for 4–8 weeks [12]. However, another meta-analysis of 17 RCTs found leucine-isolated supplementation had no significant effect on total lean mass, handgrip strength and leg press in older adults [20]. Observational studies have found that circulating BCAAs concentrations are associated with sarcopenia or muscle mass. A cross-sectional study in Norway found that the non-fasting plasma concentrations of leucine and isoleucine were significantly lower among sarcopenic participants (n = 90), compared with non-sarcopenic participants (n = 327) [21]. A cross-sectional analysis of a UK Biobank cohort study involving over 100,000 participants further identified significant positive associations between total circulating BCAA, isoleucine, leucine, valine concentrations and muscle mass [13]. A 7-year longitudinal study in Asians (n = 1140) showed that baseline circulating BCAAs were positively associated with change in skeletal muscle mass index and reduced odds of muscle mass loss among patients with type 2 diabetes [22].

Dietary BCAA intake has received less attention compared to BCAA supplementation and circulating BCAAs. The report of a joint WHO/FAO/UNU expert consultation recommends that the estimated average requirements for leucine, isoleucine and valine in adults are 39 mg, 20 mg and 26 mg per kilogram of body weight per day, respectively [23]. In this study, the proportions of participants failing to meet the EAR for leucine, isoleucine, and valine were 10.8%, 6.4%, and 9.1%, respectively. We observed a continuous upward trend in the prevalence of inadequate BCAA intake, indicating that individuals are less likely to obtain sufficient BCAAs from their diet. Although a recent study has found that there was no significant correlation between dietary intake of BCAA and its plasma concentration [24], several other studies have reported associations between dietary BCAA intake and both muscle mass [25,26] and handgrip strength [27], with relatively small sample sizes. Our findings are consistent with previous studies reporting positive associations between BCAA intake and muscle mass or handgrip strength. To our knowledge, this study is the first to identify a significant association between dietary BCAA intake and ASM within a large-scale Chinese population. Notably, our findings suggest that the beneficial effect of BCAA on ASM becomes more pronounced with increasing age. This implies a compensatory mechanism of dietary BCAA against age-related muscle loss, which may be particularly important in individuals aged 53 years when the decline in ASM accelerates. However, we did not observe the effect of physical activity on handgrip strength. This may be attributable to the differential effects of physical activity types: resistance training exerts a robust influence on muscle strength, whereas other physical activities may have a limited impact on muscle strength development. The assessment of physical activity in this study did not allow for differentiation between resistance training and other forms of activity, which may have attenuated the observed association. Therefore, the lack of a strong relationship between physical activity and handgrip strength in this study should be interpreted with caution.

In addition, we identified a nonlinear dose–response relationship between dietary BCAA intake and handgrip strength, characterized by an inverted U-shaped association. An approximate intake of 6 g/d/1000 kcal may represent the optimal range for supporting muscular function. The relatively lower effect observed in the Q5 compared with Q4 suggests that excessive BCAA intake may not confer additional benefits. One possible explanation is that high levels of BCAA intake may be associated with adverse metabolic effects, such as impaired insulin sensitivity. Evidence suggests that BCAA metabolism is closely linked to metabolic health, and elevated circulating BCAA levels are associated with insulin resistance [28]. Animal studies indicate that excessive BCAA exposure impairs insulin signaling through pathways such as mTORC1 activation and alters energy metabolism [29,30]. Notably, some recent evidence indicates that alterations in BCAA metabolism do not necessarily lead to insulin resistance, highlighting the complexity and potential bidirectional nature of this relationship [31]. In contrast, evidence specifically addressing dietary BCAA intake in human populations remains limited and inconsistent [32]. Therefore, the link between BCAAs and insulin resistance is biologically plausible but remains complex and incompletely understood in human populations, particularly with respect to dietary intake.

The present study found that the association between dietary intake of BCAA and handgrip strength was significantly affected by gender and age. In the gender-stratified analysis, males showed a strong association between dietary intake of BCAA and handgrip strength compared to females. We did not observe a significant association between dietary intake of BCAA and handgrip strength in fully adjusted models among females. Our results are consistent with those of a recent study [13]. This discrepancy between genders can be attributed several factors, including muscle mass, levels of circulating testosterone [13], skeletal muscle protein fractional synthesis rate [33], and skeletal muscle kinetics and fiber-type composition [34]. In the age-stratified analysis, when fully adjusted, we found that a moderate BCAA intake was sufficient to benefit younger adults, while only Q4 was associated with improved handgrip strength among older adults. This observation aligns with the phenomenon of anabolic resistance, a characteristic feature of sarcopenia in which aging muscle displays diminished protein synthetic responses to submaximal amino acid provision [35]. The higher coefficient observed in the older group further implies that, when adequate intake is achieved, the functional gain may be more pronounced due to the greater room for improvement in sarcopenic muscle [36]. These findings underscore that older adults may require a higher BCAA threshold to maintain muscle strength and gain more benefits from dietary BCAA intake than younger adults. However, replication in prospective cohorts and intervention trials is needed to establish causality and define optimal intake levels.

The AWGS 2025 consensus formally endorses multimodal exercise interventions with nutritional optimization (adequate protein intake supplemented with BCAA when needed) [6]. Although the latest consensus primarily emphasizes the importance of BCAA as a nutritional supplement, the contribution of dietary sources of BCAA should also be of concern. Analysis of the dietary sources of BCAA reveals that overall BCAA intake has increased in recent years, with a rising proportion derived from animal-based foods (meat, poultry, milk, and eggs) and a declining proportion from plant-based sources (cereals, tubers and legumes). Furthermore, research has found that BCAA from grain sources shows a negative association with handgrip strength, whereas BCAA from non-grain sources exhibits a positive association [27]. However, as significant non-grain sources, the consumption of red meat and processed red meat is associated with a higher risk of cardiovascular diseases and diabetes [37]. Most healthy dietary patterns recommend limiting the intake of red meat and processed red meat [38]. Legumes are considered a high-quality source of protein. With adequate intake, their effectiveness in promoting muscle synthesis and growth is equivalent to that of animal protein [39]. Additionally, they offer benefits such as improved cardiovascular health and antioxidant properties [40]. Therefore, dietary diversity of BCAA sources and a higher legume intake may be helpful for improving handgrip strength among Chinese adults.

This study has several strengths. First, the analysis utilized the longitudinal data from CHNS, which provides a substantial sample size and enhances the generalizability of the findings. Second, the employment of the mixed-effects models effectively accounted for the correlation inherent in repeated measurements and adjusted for inter-individual variability. Third, this study provides a comprehensive assessment of muscle by concurrently investigating both ASM and handgrip strength, offering a more holistic perspective.

There are several limitations. First, although based on longitudinal data, the study design did not explore the causality between dietary intake of BCAA and ASM/handgrip strength. Second, dietary BCAA intake may be subject to measurement error, attributed to: (1) recall bias, intra-individual variability, and underreporting inherent to the three 24 h dietary recalls; (2) the imputation of BCAA content for numerous foods using data from comparable food items; (3) no information about BCAA supplements. ASM may also be subject to measurement error because the muscle mass was measured using BIA (may be affected by hydration status) rather than DEXA (the gold standard). Third, handgrip strength was measured only in the last wave (2022–2024), limiting interpretation to a cross-sectional analysis. Future research should consider longitudinal association between dietary BCAA intake and handgrip strength, the need for interventional studies, and can incorporate quality of life to evaluate the health consequences of sarcopenia, and objective biomarkers to improve dietary assessment accuracy.

## 5. Conclusions

The positive associations between dietary intake of BCAA and ASM/handgrip strength were observed among 36,086 Chinese adults. The beneficial effect of dietary intake of BCAA on ASM is more significant in middle-aged and older adults. The association between BCAA intake and handgrip strength is particularly evident in males and older-age groups. It is recommended that Chinese residents diversify their dietary sources of BCAA.

## Figures and Tables

**Table 1:** Characteristics of participants by CHNS waves (2015, 2018 and 2024).

Variable	Total (n = 36,086)	2015 (n = 12,609)	2018 (n = 11,417)	2024 (n = 12,060)	*p*
Age, years	54.32 ± 14.63	52.51 ± 14.62	54.88 ± 14.32	55.67 ± 14.74	<0.001
Gender (*n*, %)					0.002
Male	15,587 (43.19)	5607 (44.47)	4861 (42.58)	5119 (42.45)	
Female	20,499 (56.81)	7002 (55.53)	6556 (57.42)	6941 (57.55)	
Residence (*n*, %)					<0.001
Urban	13,029 (36.11)	4748 (37.66)	4216 (36.93)	4065 (33.71)	
Rural	23,057 (63.89)	7861 (62.34)	7201 (63.07)	7995 (66.29)	
Education level (*n*, %)					<0.001
Middle school or below	24,000 (66.51)	8294 (65.78)	7643 (66.94)	8063 (66.86)	
High school	7147 (19.81)	2661 (21.10)	2364 (20.71)	2122 (17.60)	
College or above	4939 (13.69)	1654 (13.12)	1410 (12.35)	1875 (15.55)	
BCAA (g/d/1000 kcal)	5.31 ± 1.55	5.22 ± 1.42	5.23 ± 1.47	5.48 ± 1.73	<0.001
Not meeting EAR (*n*, %)					
Leucine	3888 (10.78)	1213 (9.62)	1200 (10.51)	1475 (12.23)	
Isoleucine	2324 (6.44)	685 (5.43)	690 (6.04)	949 (7.88)	
Valine	3279 (9.09)	985 (7.81)	961 (8.42)	1333 (11.05)	
ASM (kg)	18.68 ± 4.21	18.58 ± 4.16	18.58 ± 4.25	18.87 ± 4.23	<0.001
BMI, (kg/m^2^)	24.21 ± 3.22	23.99 ± 3.21	24.24 ± 3.20	24.43 ± 3.22	<0.001
Energy (kcal)	1989.41 ± 749.05	2024.40 ± 738.02	2022.84 ± 742.23	1921.17 ± 762.22	<0.001
Protein (g/d)	64.22 ± 26.46	65.09 ± 26.27	64.93 ± 26.31	62.63 ± 26.73	<0.001
Fat (g/d)	83.67 ± 47.08	81.01 ± 43.55	80.50 ± 46.10	89.46 ± 50.87	<0.001
Carbohydrate (g/d)	240.22 ± 108.09	253.75 ± 110.32	254.95 ± 105.82	212.13 ± 102.21	<0.001
Physical activity MET · h/week	141.02 ± 135.77	141.43 ± 142.82	158.46 ± 151.63	124.07 ± 107.20	<0.001
Alcohol use (*n*, %)	9609 (26.63)	3461 (27.45)	2779 (24.34)	3369 (27.94)	<0.001
Smoking (*n*, %)					<0.001
Never	27,273 (75.58)	9343 (74.10)	8828 (77.32)	9102 (75.47)	
Quit	1207 (3.34)	378 (3.00)	294 (2.58)	535 (4.44)	
Current smoker	7606 (21.08)	2888 (22.90)	2295 (20.10)	2423 (20.09)	
Type 2 diabetes (*n*, %)	4930 (13.66)	1464 (11.61)	1578 (13.82)	1888 (15.66)	<0.001
Handgrip strength (kg)		Not measured	Not measured	29.66 ± 9.89	

Abbreviations: CHNS, China Health and Nutrition Survey; BCAA, branched-chain amino acid; EAR, estimated average requirement; ASM, appendicular skeletal muscle mass; BMI, body mass index; MET, metabolic equivalents of task.

**Table 2:** Mixed-effects estimate on ASM among Chinese adults.

Variable	Model 1		Model 2		Model 3	
	β (95%CI)	*p*	β (95%CI)	*p*	β (95%CI)	*p*
BCAA	0.070 (0.054, 0.086)	<0.001	0.078 (0.061, 0.095)	<0.001	0.074 (0.058, 0.090)	<0.001
Age	−0.007 (−0.009, −0.005)	<0.001	−0.011 (−0.013, −0.009)	<0.001	−0.011 (−0.013, −0.009)	<0.001
Age squared	−0.001 (−0.001, −0.001)	<0.001	−0.001 (−0.001, −0.000)	<0.001	−0.001 (−0.001, −0.000)	<0.001
Gender						
Male	REF		REF		REF	
Female	−6.558 (−6.627, −6.489)	<0.001	−6.548 (−6.607, −6.489)	<0.001	−6.563 (−6.635, −6.491)	<0.001
BMI			0.379 (0.371, 0.387)	<0.001	0.379 (0.370, 0.387)	<0.001
BCAA * age			0.001 (−0.000, 0.001)	0.281	0.001 (−0.000, 0.001)	0.247
BCAA * age squared			−0.000 (−0.000, 0.000)	0.841	−0.000 (−0.000, 0.000)	0.868
BCAA * gender			−0.027 (−0.057, 0.003)	0.075	−0.026 (−0.055, 0.004)	0.088
Educational level						
Middle school or below			REF		REF	
High school			0.190 (0.120, 0.260)	<0.001	0.190 (0.118, 0.259)	<0.001
College or above			0.397 (0.309, 0.485)	<0.001	0.395 (0.303, 0.480)	<0.001
Residence						
Urban			REF		REF	
Rural			−0.163 (−0.225, −0.100)	<0.001	−0.163 (−0.225, −0.100)	<0.001
Alcohol use					0.008 (−0.055, 0.070)	0.792
Smoking						
Never					REF	
Quit					0.065 (−0.065, 0.194)	0.330
Current smoker					−0.052 (−0.128, 0.025)	0.185
Physical activity						
Low					REF	
High					0.020 (0.008, 0.032)	<0.001
BCAA * physical activity					0.006 (−0.023, 0.036)	0.652

Abbreviations: BCAA, branched-chain amino acid; ASM, appendicular skeletal muscle mass; BMI, body mass index; * Statistical significance at *p* < 0.05.

**Table 3:** Multivariable regression analysis between BCAA intake and handgrip strength among participants in CHNS 2024.

	Model 1		Model 2		Model 3	
	β-Coefficient ± SE	*p*	β-Coefficient ± SE	*p*	β-Coefficient ± SE	*p*
All participants						
Q1	REF		REF		REF	
Q2	0.734 ± 0.285	0.010	0.528 ± 0.212	0.013	0.499 ± 0.211	0.018
Q3	0.539 ± 0.285	0.059	0.463 ± 0.213	0.030	0.439 ± 0.212	0.039
Q4	0.901 ± 0.285	0.002	0.766 ± 0.214	<0.001	0.721 ± 0.214	<0.001
Q5	0.667 ± 0.285	0.019	0.416 ± 0.216	0.055	0.372 ± 0.219	0.090
Male						
Q1	REF		REF		REF	
Q2	1.146 ± 0.406	0.005	1.002 ± 0.363	0.006	0.948 ± 0.359	0.008
Q3	0.789 ± 0.406	0.052	0.617 ± 0.364	0.090	0.599 ± 0.361	0.098
Q4	1.169 ± 0.406	0.004	1.074 ± 0.370	0.003	1.016 ± 0.365	0.005
Q5	1.135 ± 0.406	<0.001	0.727 ± 0.371	0.050	0.717 ± 0.373	0.055
Female						
Q1	REF		REF		REF	
Q2	0.102 ± 0.259	0.693	0.117 ± 0.248	0.638	0.073 ± 0.248	0.768
Q3	0.475 ± 0.259	0.067	0.306 ± 0.249	0.219	0.244 ± 0.249	0.327
Q4	0.662 ± 0.259	0.011	0.505 ± 0.250	0.044	0.413 ± 0.251	0.100
Q5	0.473 ± 0.259	0.067	0.160 ± 0.252	0.527	0.054 ± 0.258	0.833
<65 years						
Q1	REF		REF		REF	
Q2	0.894 ± 0.342	0.009	0.568 ± 0.252	0.024	0.517 ± 0.250	0.039
Q3	0.474 ± 0.342	0.166	0.688 ± 0.253	0.007	0.575 ± 0.252	0.023
Q4	0.623 ± 0.342	0.068	0.666 ± 0.255	0.009	0.511 ± 0.255	0.044
Q5	0.384 ± 0.342	0.261	0.622 ± 0.257	0.015	0.358 ± 0.262	0.159
≥65 years						
Q1	REF		REF		REF	
Q2	0.537 ± 0.454	0.237	0.620 ± 0.383	0.105	0.507 ± 0.378	0.180
Q3	0.006 ± 0.454	0.990	0.050 ± 0.384	0.896	−0.136 ± 0.381	0.722
Q4	1.509 ± 0.454	<0.001	1.456 ± 0.386	<0.001	1.024 ± 0.386	0.008
Q5	0.486 ± 0.454	0.285	0.970 ± 0.388	0.012	0.494 ± 0.390	0.206

Abbreviations: CHNS, China Health and Nutrition Survey; SE, standard error.

## Data Availability

The data presented in this study are available on request from the corresponding author.

## Supplementary Materials

The following supporting information can be downloaded at: https://www.mdpi.com/article/10.3390/nu18101546/s1, Figure S1: The proportion of dietary BCAA intake from different food sources across CHNS 2015–2024; Figure S2: RCS analysis of association between BCAA intake and ASM among males (A), females (B), <65 years group (C) and ≥65 years group (D). Table S1: Mixed-effects estimate on ASM among Chinese adults without diabetes. Figure S3. RCS analysis of association between BCAA intake and handgrip strength among participants without diabetes.

## Abbreviations

The following abbreviations are used in this manuscript:

BCAAs	Branched-chain amino acids
ASM	Appendicular skeletal muscle mass
CHNS	China Health and Nutrition Survey
AWGS	Asian Working Group for Sarcopenia
DEXA	Dual-energy X-ray absorptiometer
BIA	Bioelectrical impedance analysis
mTORC1	Mammalian target of rapamycin complex 1
RCTs	Randomized controlled trials
EAR	Estimated average requirements
T2DM	Type 2 diabetes mellitus
MET	Metabolic equivalents of task
RCS	Restricted cubic spline
SD	Standard deviation
SE	Standard error
CI	Confidence interval
