# Supplementary material for: Dietary Branched-Chain Amino Acid Intake Is Associated with Muscle Mass and Handgrip Strength: Evidence from China—Health and Nutrition Survey 2015–2024
Source: Nutrients. 2026 May 13;18(10):1546. doi: 10.3390/nu18101546 (PMC13209909; doi:10.3390/nu18101546)
Supplement: Supplementary file 1 [file nutrients-18-01546-s001.zip › nutrients-4270156-supplementary.pdf]

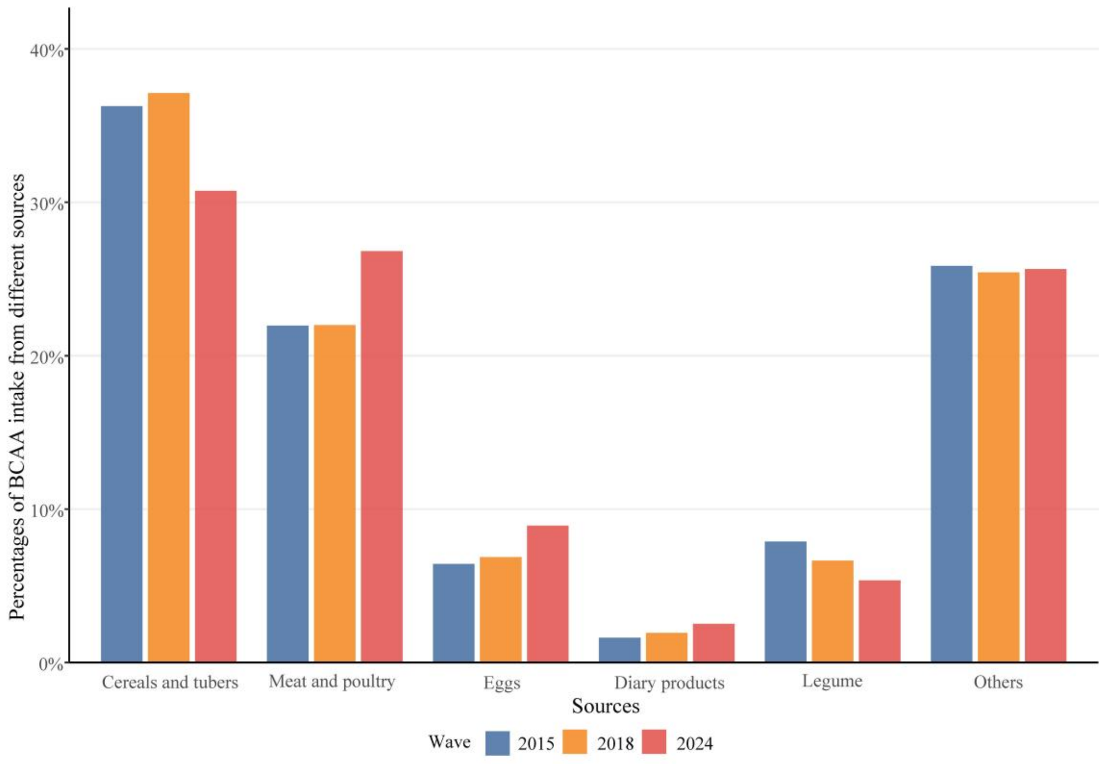

Supplementary Figure S1. The proportion of dietary BCAA intake from different food sources across CHNS 2015-2024

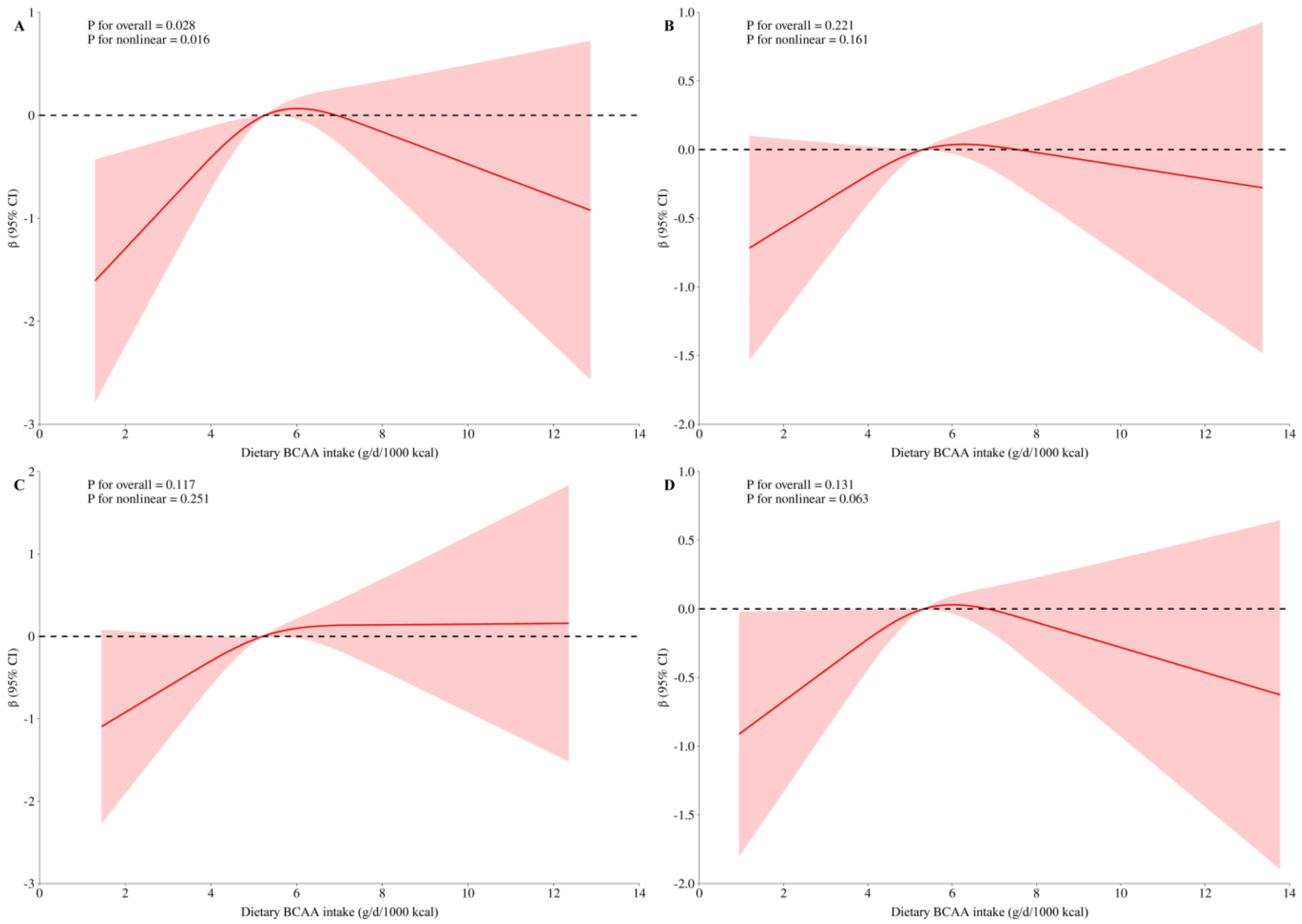

Supplementary Figure S2. RCS analysis of association between BCAA intake and handgrip strength among males (A), females (B), <65 years group (C) and ≥65 years group (D).

Supplementary Table S1. Mixed effects estimate on ASM among Chinese adults without

Variable	Model 1		Model 2		Model 3	
	β (95%CI)	P	β (95%CI)	P	β (95%CI)	P
BCAA	0.073 (0.056, 0.090)	<0.001	0.122 (0.067, 0.176)	<0.001	0.111 (0.053, 0.169)	<0.001
Age	-0.010 (-0.012, -0.007)	<0.001	-0.012 (-0.014, -0.010)	<0.001	-0.012 (-0.014, -0.010)	<0.001
Age-squared	-0.001 (-0.001, -0.001)	<0.001	-0.001 (-0.001, -0.000)	<0.001	-0.001 (-0.001, -0.000)	<0.001
Gender						
Male	REF		REF		REF	
Female	-6.513 (-6.586, -6.440)	<0.001	-6.494 (-6.556, -6.432)	<0.001	-6.503 (-6.580, -6.426)	<0.001
BMI			0.380 (0.371, 0.389)	<0.001	0.380 (0.371, 0.389)	<0.001
BCAA* age			0.001 (-0.000, 0.001)	0.246	0.001 (-0.000, 0.002)	0.168
BCAA* Age-squared			-0.000 (-0.000, 0.000)	0.796	-0.000 (-0.000, 0.000)	0.87
BCAA* Gender			-0.037 (-0.069, -0.005)	0.023	-0.036 (-0.068, -0.004)	0.027
Educational level						
Middle school or below			REF		REF	
High school			0.228 (0.153, 0.304)	<0.001	0.227 (0.152, 0.302)	<0.001
College or above			0.438 (0.346, 0.529)	<0.001	0.433 (0.340, 0.525)	<0.001
Residence						
Urban			REF		REF	
Rural			-0.147 (-0.214, -0.080)	<0.001	-0.147 (-0.214, -0.081)	<0.001
Alcohol use					0.027 (-0.040, 0.094)	0.434
Smoking						
Never					REF	
Quit					0.022 (-0.125, 0.169)	0.771
Current smoker					-0.052 (-0.134, 0.031)	0.22
Physical activity						
Low					REF	
High					0.011 (-0.038, 0.060)	0.657
BCAA* Physical activity					0.015 (-0.016, 0.046)	0.335

diabetes.

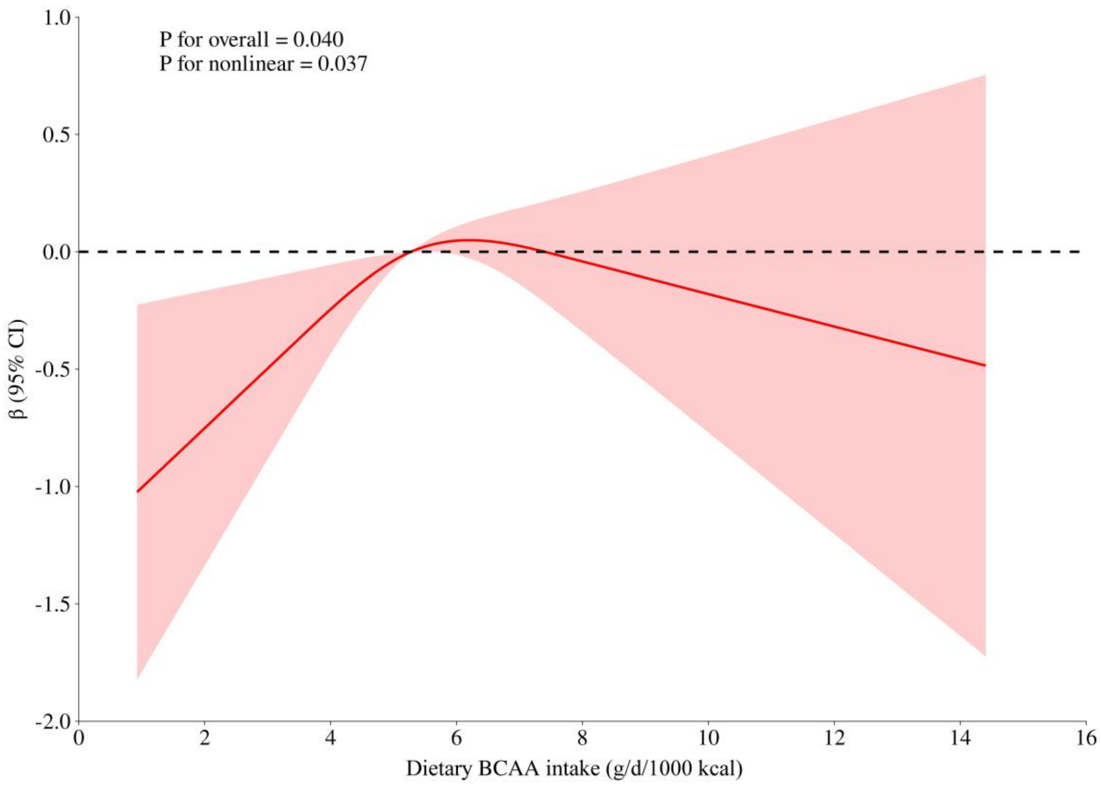

Supplementary Figure S3. RCS analysis of association between BCAA intake and handgrip strength among participants without diabetes.
